# Thermodynamic effects of gas adiabatic index on cavitation bubble collapse

**DOI:** 10.1016/j.heliyon.2023.e20532

**Published:** 2023-10-12

**Authors:** Yu Yang, Minglei Shan, Xuefen Kan, Kangjun Duan, Qingbang Han, Yue Juan

**Affiliations:** a College of Information Science and Technology, Nanjing Forestry University, Nanjing 210037, China; b Jiangsu Key Laboratory of power Transmission and Distribution Equipment Technology, Hohai University, Changzhou 213022, China; c Department of Transportation Engineering, Jiangsu Shipping College, Nantong 226000, China; d Helmholtz Institute Ulm, Karlsruhe Institute of Technology, Ulm, 89081, Germany

**Keywords:** Multicomponent, Lattice Boltzmann method, cavitation bubble, Gas properties, Adiabatic index

## Abstract

In this paper, an improved multicomponent lattice Boltzmann model is employed to investigate the impact of the gas properties, specifically the gas adiabatic index, on the thermodynamic effects of cavitation bubble collapse. The study focuses on analyzing the temperature evolution in the flow field and the resulting thermal effects on the surrounding wall. The accuracy of the developed model is verified through comparisons with analytical solutions of the Rayleigh-Plesset equation and the validation of the adiabatic law. Then, a thermodynamic model of cavitation bubble composed of two-mixed gases collapsing near a wall is established to explore the influence of the gas adiabatic index *γ* on the temperature behavior. Key findings include the observation that the *γ* affects the temperature of the first collapse significantly, while its influence on the second collapse is minimal. Additionally, the presence of low-temperature regions near the bubble surface during collapse impacts both bubble and wall temperatures. The study also demonstrates that the *γ* affects maximum and minimum wall temperatures. The results have implications for selecting specific non-condensable gas properties within cavitation bubbles for targeted cooling or heating purposes, including potential applications in electronic component cooling and environmental refrigeration.

## 1 Introduction

Cavitation bubble contains astonishing energy. It can be regarded as a complex system composed of multiple gases and is widely used in biomedical, protein degradation, surface technology, and other fields [[1], [2], [3], [4]]. Research has clarified that gas properties affect the thermodynamic processes of cavitation bubble collapse. Especially, gas adiabatic index is an essential physical quantity to describe the properties of gases. It will affect the internal energy of gas, the internal motion of gas molecules, and the application of thermal engineering technology [[5], [6], [7]].

As early as 1953, Peterka [8] investigated the influence of non-condensable gases on cavitation. It is demonstrated that an explosion sound and hammering sound generated by cavitation erosion in the pipe will be reduced with the increase of air content through the experiment of cavitation erosion reduction. Later, Song et al. [9] analyzed the influence of non-condensable gases content on the growth and development of cavitation. Qiang et al. [10] studied the influence of non-condensable gases mass fraction in a bubble on local cavitation flow in a natural cavitation model of underwater aviation. Although the above scholars have studied the effects of non-condensable gases on the cavitation initiation and collapse process, there is still a lack of relevant conclusions on the thermodynamic properties of the bubble. The adiabatic index is a significant thermodynamic parameter of gases, reflecting the change in internal energy during compression and expansion processes. Different types of gases possess distinct adiabatic indices, which are contingent upon the molecular structure and vibration modes of the gas. Some non-condensable gases in nature -- monatomic gases such as He, Ne, Ar, Ke and other rare gases; Diatomic gas, such as H_2_, N_2_, O_2_ etc.; Inorganic gases, such as polyatoms such as CO_2_, SO_2_ have different adiabatic indexes, which will affect their thermodynamic properties, and finally affect the cavitation effect [11]. A higher adiabatic index signifies a lesser change in internal energy during compression, leading to a temperature rise. Therefore, the investigation of gas adiabatic index is of great significance for studying the properties of gases and determining the phase transition.

However, considering factors such as energy transfer inside the non-condensable gas, hydrodynamic interaction, and liquid-solid heat transfer will increase the complexity and unpredictability of cavitation. Existing experimental methods are unable to satisfy the requirements for accurate measurement of cavitation bubble temperature. It is necessary to explore its thermodynamic effect mechanism by numerical simulation. Computational Fluid Dynamics (CFD) is a field that utilizes computer simulations and numerical methods to study phenomena related to fluid motion, heat transfer, mass transfer, and more. It has been successfully applied to research in the field of cavitation. Traditional CFD methods include the finite difference method (FVM), finite element method (FEM), and finite volume method (FVM), which are capable of solving compressible and incompressible flows, two-phase potential flows, viscous flows, and more. However, these methods also face challenges such as numerical instability, grid dependence, model uncertainties, and the need for interface capturing or tracking algorithms, which can limit computational efficiency, particularly in multi-component and multiphase flow simulations. In comparison to traditional CFD methods, the lattice Boltzmann method (LBM), based on a mesoscopic approach, can conveniently describe fluid–fluid or fluid–solid interactions conveniently [[12], [13], [14], [15]]. It has charming advantages and potential in dealing with complex flows under the coupling conditions of multi-scale and multi-physical field [[16], [17], [18], [19]]. Mesoscopic LBM has attracted much attention due to its clear physical background, easy program parallel implementation, simple algorithm and flexible multicomponent multiphase (MCMP) boundary processability [20]. Meantime, LBM has clear particle motion images, which can easily handle and depict the interaction between fluid and the surrounding environment, and can successfully simulate MCMP fluid.

The first MCMP pseudopotential model was proposed by Shan and Doolen [21]. It uses the force between particles to control the interaction intensity of the same and different components. However, the origin MCMP pseudopotential model has several defects, such as large virtual velocity, low density and kinematic viscosity ratio, etc. Therefore, many scholars later proposed many improved methods to overcome the above problem. For example, Bao and Schaefer [22] introduced appropriate equation of state (EOS) to achieve the density ratio and the better numerical stability; Hou et al. [23] adopted an external force term into the control equation to expand the limits of density ratio and viscosity ratio; He et al. [24] used MRT collision operator to achieve the same large density ratio. Many efforts make LBM simulation results closer to the actual physical phenomena of the MCMP flow [[25], [26], [27]].

Along with the MCMP flow model, the thermal model is also developed. Many thermal LBM models have been developed since 1993, which reflect the basic characteristics of thermal flow and have higher calculation accuracy, better numerical stability and simple algorithm structure [[28], [29], [30]]. Thermal LBM models can be roughly divided into the multispeed model [31,32], double distribution function (DDF) model [33,34], and hybrid model combined with difference method [35,36]. The DDF lattice Boltzmann (LB) model is composed of two kinds of distribution functions, which has good numerical stability. Zhang and Chen firstly proposed the DDF thermal LBM based on the pseudopotential [37]. Then, Gong et al. [38], Biferale et al. [39], and Házi et al. [40,41] also proposed other forms of thermal LB model. The common features of these models are that *g*(*x*) is used to recover the macroscopic temperature equation and simulate the temperature field at Naiver-Stocks (N–S) level. Besides, *f*(*x*) and *g*(*x*) are coupled by a temperature term. The model proposed by Gong et al. can provide more accurate numerical result, and greatly reduce the generation of virtual velocity. These models can capture the details of phase interface dynamics and provide a more microscopic view of two-phase flow. On the basis of the above research, Li et al. [42] developed a thermal multi-relaxation-time (MRT) LBM to simulate thermal multiphase flow. The improved model can eliminate the discrete effect of temperature source term, which is greatly improved the numerical stability.

By the improvement and optimization of thermal model, it can be more widely used in the field of phase change heat transfer. Cavitation, as one of the hot topics in the multiphase flow field, is usually accompanied by a variety of complex macroscopic dynamic. In terms of research on cavitation bubble thermodynamics, Yang et al. first established a thermodynamic model of cavitation bubble collapsing by using the improved thermal DDF-LBM [43,44]. Moreover, a multicomponent DDF LBM was developed on this basis, and the validity of the model was verified by theoretical and experimental comparison, which can be used for the thermodynamic study of multicomponent multiphase flow [45]. This work assumes the presence of two gas components, i.e., non-condensable gas and water vapor, inside the cavitation bubble. Based on the aforementioned coupled model of multicomponent pseudopotential MRT LBM and thermal multicomponent MRT LBM, the thermodynamics of the gas-liquid phase transition involving two gas components are simulated. The primary focus is on investigating the influence of non-condensable gas properties on the temperature of the bubble and its thermal effect.

The overall arrangement of this work is as follows. The multicomponent LBM and the description of lattice units are introduced in Section 2. The radius and temperature verification of spherical cavitation bubble is given in Section 3. The influence of gas properties on thermal effect of cavitation bubble is discussed in Section 4. Section 5 is arranged as the conclusion and discussion.

## 2 Multicomponent lattice Boltzmann methods

In the real physical world, there are multiple gas components inside cavitation bubbles. The use of a multi-component multiphase LB model allows for the simulation of the evolution and interactions of different components. A pseudopotential multicomponent multiphase LB model and a multi-component thermal lattice model with the MRT collision operator are introduced in section 2. The feasibility of the proposed model for two-component numerical simulation is verified.

### 2.1 Multicomponent pseudopotential MRT LBM

The particle evolution equation can be expressed by the multicomponent density distribution function, as follows [46,47].

(1) $$f_{i,\sigma }\left(x+e_{i}\delta_{t},t+\delta_{t}\right)-f_{i,\sigma }\left(x,t\right)=-M^{-1}\Lambda_{\sigma }M\left(f_{j,\sigma }\left(x,t\right)-f_{j,\sigma }^{eq}\left(x,t\right)\right)+\delta tS_{i,\sigma }^{\prime }\left(x,t\right)\text{,}$$

where $f_{i,\sigma }\left(x,t\right)$ is the density distribution function of σ−th component, ***x*** refers particle position, $\delta_{t}$ is the time step, and i is the number of discrete velocity for D2Q9 lattice model. The forcing term is $S_{i,\sigma }^{\prime }$, Λ represents the relaxation diagonal matrix, which can be written

(2) $$\Lambda =diag\left(\tau_{\rho }^{-1},\tau_{e}^{-1},\tau_{\xi }^{-1},\tau_{j}^{-1},\tau_{q}^{-1},\tau_{j}^{-1},\tau_{q}^{-1},\tau_{v}^{-1},\tau_{v}^{-1}\right)$$

where $\tau_{\rho }^{-1},\tau_{e}^{-1},\tau_{\xi }^{-1},\tau_{j}^{-1},\tau_{q}^{-1}$ and $\tau_{v}^{-1}$ represent density, energy, square root of energy, of momentum flux component, energy flow component and pressure tensor, respectively. In this work, the relaxation factor $\tau_{\rho }^{-1}=\tau_{e}^{-1}=\tau_{\xi }^{-1}=0.8,\tau_{j}^{-1}=\tau_{v}^{-1}=1.0,\tau_{q}^{-1}=1.1$.

M is the transformation matrix [48]

(3) $$M=\left[\begin{array}{ccccccccc} 1 & 1 & 1 & 1 & 1 & 1 & 1 & 1 & 1\\ -4 & -1 & -1 & -1 & -1 & 2 & 2 & 2 & 2\\ 4 & -2 & -2 & -2 & -2 & 1 & 1 & 1 & 1\\ 0 & 1 & 0 & -1 & 0 & 1 & -1 & -1 & 1\\ 0 & -2 & 0 & 2 & 0 & 1 & -1 & -1 & 1\\ 0 & 0 & 1 & 0 & -1 & 1 & 1 & -1 & -1\\ 0 & 0 & -2 & 0 & 2 & 1 & 1 & -1 & -1\\ 0 & 1 & -1 & 1 & -1 & 0 & 0 & 0 & 0\\ 0 & 0 & 0 & 0 & 0 & 1 & -1 & 1 & -1 \end{array}\right]$$

Eq. (1) can be derived from the macroscopic N–S equation. Using the transformation matrix, the particle collision step is

(4) $$m_{\sigma }^{*}=m_{\sigma }-\Lambda \left(m_{\sigma }-m_{\sigma }^{eq}\right)+\delta_{t}\left(I-\frac{\Lambda }{2}\right)S$$

where $m_{\sigma }^{*}=\left(m_{\sigma ,0}^{*},\ldots ,m_{\sigma ,i}^{*}\right)$, I refers to the unit tensor, and $S=MS^{\prime }$ is the forcing term in the moment space. The streaming step of the multicomponent equation can be expressed as

(5) $$f_{i,\sigma }\left(x+e_{i}\delta_{t},t+\delta_{t}\right)=f_{i,\sigma }^{*}\left(x,t\right)$$

where $f_{i,\sigma }^{*}=M^{-1}m_{i,\sigma }^{*}$.

The moment spaces of σ−th component is

(6) $$m_{\sigma }=Mf_{\sigma }={M_{ij}}_{,\sigma }{f_{j}}_{,\sigma }={\left(\rho_{\sigma },e_{\sigma },\varsigma_{\sigma },j_{x,\sigma },q_{x,\sigma },j_{y,\sigma },q_{y,\sigma },p_{xx,\sigma },p_{xy,\sigma }\right)}^{Τ}$$

(7) $${m_{\sigma }}^{eq}=M{f_{\sigma }}^{eq}={M_{ij}}_{,\sigma }f_{j,\sigma }^{eq}={\left(\rho_{\sigma },{e_{\sigma }}^{eq},{\varsigma_{\sigma }}^{eq},j_{x,\sigma },q_{x,\sigma }^{eq},j_{y,\sigma },q_{y,\sigma }^{eq},p_{xx,\sigma }^{eq},p_{xy,\sigma }^{eq}\right)}^{Τ}=\rho_{\sigma }{\left(1,-2+3{\left|v\right|}^{2},1-3{\left|v\right|}^{2},v_{x},-v_{x},v_{y},-v_{y},v_{x}^{2}-v_{y}^{2},v_{x}v_{y}\right)}^{Τ}$$

The macroscopic density $\rho_{\sigma }$ can be calculated by $\rho_{\sigma }=\sum_{i}f_{i,\sigma }$. The velocity $v_{\sigma }$ shows as

(8) $$v_{\sigma }=\frac{\sum_{i}e_{i}f_{i,\sigma }+\frac{\delta_{t}}{2}F_{\sigma }}{\sum_{\sigma }\rho_{\sigma }}$$

the macroscopic mixed fluid density *ρ* is the sum of *ρσ*. Thus, the modified macroscopic velocity **v** is

(9) $$v=\frac{\sum_{\sigma }\left(\sum_{\sigma }f_{i,\sigma }e_{i}+0.5F_{\sigma }\right)}{\rho }$$

where $F_{\sigma }=\left(F_{x,\sigma },F_{y,\sigma }\right)$ is the total multicomponent fluid force, expressed by Eq. (10), which includes intramolecular force and intermolecular force.

$$F_{\sigma }\left(x\right)=-G_{\sigma \sigma }\psi_{\sigma }\left(x\right)\sum_{\alpha }\omega \left(\left|e_{i}^{2}\right|\right)\psi_{\sigma }\left(x+e_{i}\delta_{t}\right)e_{i}$$

(10) $$-G_{\sigma \bar{\sigma }}\psi_{\sigma }\left(x\right)\sum_{\alpha }\omega \left(\left|e_{i}^{2}\right|\right)\psi_{\bar{\sigma }}\left(x+e_{i}\delta_{t}\right)e_{i}$$

where σ and $\bar{\sigma }$ represent two different components. The interaction strength *G* in the same component is negative, which shows an attraction. The interaction strength *G* between different components is positive, which means that there is a repulsive force between components. Considering the presence of two mixed gases within the cavitation bubble, they can be denoted as Component 1 and Component 2. For Component 1 (non-condensable gas), it is generally treated as an ideal gas, and the pseudopotential function is equal to its density [49]. As for Component 2 (water vapor and liquid water), different equations of state (EOS) can be introduced by pseudopotential function $\psi_{2}\left(x\right)=\sqrt{2\left(p_{EOS}-\rho c_{s}^{2}\right)/G_{22}}$ to achieve large density ratio, where *G*_22_ = −1. In this study, Carnahan-Starling (C–S) EOS is specified as the *p*_EOS_ of liquid [50]

(11) $$p_{EOS}=\rho R_{g}T\frac{1+\frac{b\rho }{4}+{\left(\frac{b\rho }{4}\right)}^{2}-{\left(\frac{b\rho }{4}\right)}^{3}}{{\left(1-\frac{b\rho }{4}\right)}^{3}}-a\rho^{2}$$

where $a=\frac{0.4963{\left(RT_{c}\right)}^{2}}{p_{c}}$, $b=\frac{0.1873RT_{c}}{p_{c}}$, *R*_*g*_ represents the gas constant. *T*_*c*_ is the critical temperature, and *p*_*c*_ is the critical pressure. In the present study, the above constants are set as a=1, b=4 and *R*_*g*_ = 1.

(12) $$S=\left[\begin{array}{c} 0\\ 6\left(v_{x}F_{x}+v_{y}F_{y}\right)+\frac{0.75\varepsilon {\left|F\right|}^{2}}{\psi^{2}\delta_{t}\left(\tau_{e}-0.5\right)}\\ -6\left(v_{x}F_{x}+v_{y}F_{y}\right)-\frac{0.75\varepsilon {\left|F\right|}^{2}}{\psi^{2}\delta_{t}\left(\tau_{\varsigma }-0.5\right)}\\ F_{x}\\ -F_{x}\\ F_{y}\\ -F_{y}\\ 2\left(v_{x}F_{x}-v_{y}F_{y}\right)\\ \left(v_{x}F_{y}+v_{y}F_{x}\right) \end{array}\right]$$

The improved forcing scheme in the present model is adopted Li's force scheme [51]. According to the investigation results of thermodynamic consistency in Ref. [52], adjustable parameter ε is considered as 1.86 for the component 2. While for the component 1, the adjustable parameter is 0.

By using the force scheme improved by Li et al. and ignoring the force error, the multicomponent pseudopotential MRT LB equation can be derive the macro continuity equation through C-E expansion

$$\frac{\partial \rho }{\partial t}+\nabla \cdot \left(\rho v\right)=0\text{,}$$

(13) $$\frac{\partial \rho v}{\partial t}+\nabla \cdot \left(\rho vv\right)=-\nabla \cdot p+\nabla \cdot \Pi$$

### 2.2 Improved multicomponent thermal LBM

The target temperature equation can be written as [53,54].

(14) $$\frac{\partial T}{\partial t}+\nabla \cdot \left(vT\right)=\nabla \cdot \left(\alpha \nabla T\right)+\varphi$$

where $\alpha =\frac{k}{\rho c_{v}}$ represents the thermal diffusivity, $c_{v}$ represents the specific heat at constant volume, and k is the thermal conductivity. φ refers to the temperature source term.

Eq. (14) can be solved by the improved multicomponent thermal LBE, which is modified from single-component model [46,47]. The equation of temperature distribution function *g*(*x*) contains σ−th components is

(15) $$g_{i,\sigma }\left(x+e_{i}\delta_{t},t+\delta_{t}\right)-g_{i,\sigma }\left(x,t\right)=-{\bar{\Lambda }}_{ij,\sigma }\left(g_{j,\sigma }-g_{j,\sigma }^{eq}\right){|}_{\left(x.t\right)}+\delta_{t}Q_{i,\sigma }^{\prime }\left(x,t\right)$$

where ${\bar{\Lambda }}_{ij,\sigma }={\left(M^{-1}\Lambda_{\sigma }M\right)}_{ij}$, $Q_{i,\sigma }^{\prime }$ is the modified source term, and the lattice model adopts D2Q9. The streaming step of Eq. (15) is given by

(16) $$g_{i,\sigma }\left(x+e_{i}\delta_{t},t+\delta_{t}\right)=g_{i,\sigma }^{*}\left(x,t\right)$$

where $g_{i,\sigma }^{*}=M^{-1}n_{i,\sigma }^{*}$.

The temperature distribution function on the moment space $n_{\sigma }=Mg_{\sigma }$, and $g_{\sigma }={\left(g_{0,\sigma },g_{1,\sigma },...,g_{8,\sigma }\right)}^{Τ}$. $n_{\sigma }^{eq}$ is expressed as

(17) $$n_{\sigma }^{eq}=T_{\sigma }{\left(\begin{array}{ccccccccc} 1, & -2, & 2, & v_{x}, & -v_{x}, & v_{y}, & -v_{y}, & 0, & 0 \end{array}\right)}^{Τ}$$

where $T_{\sigma }=\sum_{i}g_{i,\sigma }$. Eq. (19) converted in moment space is as follows

(18) $${n_{\sigma }}^{*}=n_{\sigma }-\Lambda \left(n_{\sigma }-n_{\sigma }^{eq}\right)+\delta_{t}Q_{\sigma }$$

where $n_{\sigma }={\left(n_{0,\sigma }^{*},n_{1,\sigma }^{*},...,n_{8,\sigma }^{*}\right)}^{Τ}$ and $Q_{\sigma }$ represents the temperature source term in moment space

(19) $$Q_{\sigma }={\left(\begin{array}{ccccccccc} Q_{0,\sigma }, & 0, & 0, & 0, & 0, & 0, & 0, & 0, & 0 \end{array}\right)}^{Τ}$$

where $Q_{0,\sigma }=\varphi_{\sigma }+0.5\delta_{t}\partial_{t}\varphi_{\sigma }$. The source term is written as

(20) $$\varphi_{\sigma }=T_{\sigma }\left[1-\frac{1}{\rho c_{v}}{\left(\frac{\partial p_{EOS}}{\partial T_{\sigma }}\right)}_{\rho }\right]\nabla \cdot v$$

To solve the above equation, we set $\partial_{t}\varphi_{\sigma }\approx \frac{\left[\varphi_{\sigma }\left(t\right)-\varphi_{\sigma }\left(t-\delta_{t}\right)\right]}{\delta_{t}}$ in numerical implementations.

### 2.3 Description of lattice units

Table 1 shows that in order to facilitate the horizontal comparison of models established by the same modeling method, the unit of all physical quantities in this paper adopt lattice unit.

**Table 1:** Basic lattice units.

Name	Variable	Units	Dimension
**time**	*δt*	ts	T
**length**	*δx*	lu	L
**mass**	*m*	mu	M
**density**	*ρ*	mu/lu^3^	M/L^3^
**velocity**	*v*	lu/ts	L/T
**viscosity**	*μ*	lu^2^/ts	L^2^/T
**pressure**	*p*	mu·lu/ts^2^	ML/T^2^

## 3 Model validation

### 3.1 Computation domain of collapsing cavitation bubble

**Fig. 1:**
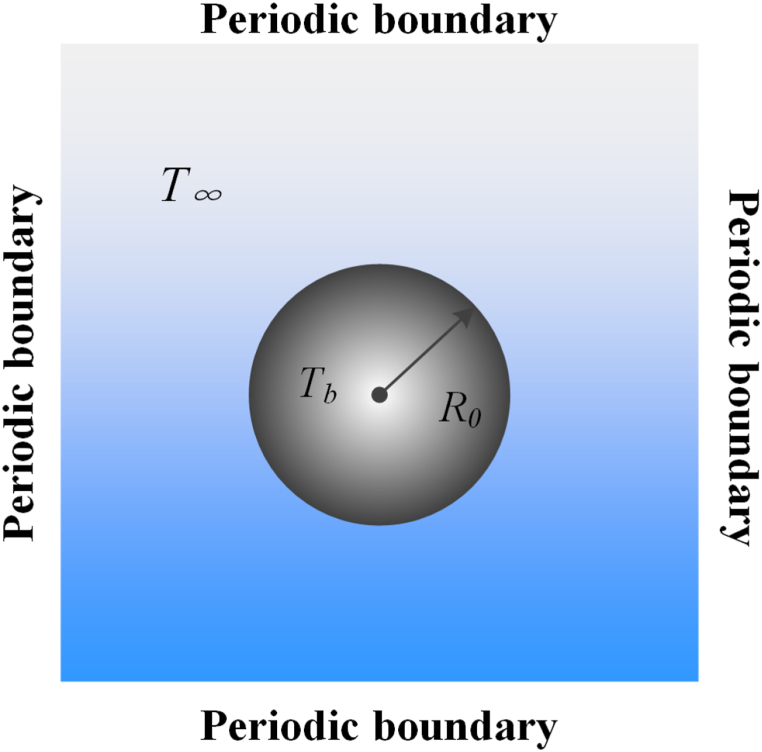
Spherical bubble model.

As shown in Fig. 1, the static spherical bubble lies at the center of an infinite liquid field without gravitational field. The spherical bubble contains two mixed gases (vapor and ideal non-condensable gas). The initial radius *R*_0_ = 60, and the numerical calculation domain is 301 × 301 lattice. Periodic boundary conditions are applied in all directions. The density initialization formula of flow field is [54].

(21) $$\rho \left(x,y\right)=\frac{\rho_{in}+\rho_{out}}{2}+\frac{\rho_{in}-\rho_{out}}{2}\times \tan\;h\left[\frac{2\left(\sqrt{{\left(x-x_{0}\right)}^{2}+{\left(y-y_{0}\right)}^{2}}-R_{0}\right)}{W}\right]$$

where $\rho_{in}$ and $\rho_{out}$ represent the density inside and outside of the bubble, respectively. $\tan\;h\left(x\right)=\left(e^{2x}-1\right)/\left(e^{2x}+1\right)$ is the hyperbolic tangent function. *W* = 5 is the width of phase interface. $\left(x_{0},y_{0}\right)$ is the central point of domain. Initially, the cavitation bubble is in equilibrium state, as shown in Fig. 1.

According the linear bubble dynamics equation, the equilibrium state of pressure inside and outside the bubble can be expressed as

(22) $$p_{l}=p_{g}+p_{v}-p_{\sigma }$$

thus, the equilibrium initial density of gas and vapor can be obtained.

In the previous work of our group [47], we studied the temperature and pressure distributions of collapsing cavitation bubble under three different volume fraction ratios of two mixed gases. The results showed that the cavitation effects are most obvious when the volume fraction ratio is 50 %. Therefore, in this work, it is assumed that the volume of two gases in the bubble is half and the molar mass of the gas is equal. Initially, the density of gas (liquid) is set as 0.000893 (0.00085) and 0.000001 (0.406) inside and outside the bubble at a dimensionless relative temperature *T*_*r*_ = 0.6*T*_*c*_, respectively. Thus, Eq. (22) can be satisfied, and the bubble is in equilibrium. The cavitation bubble temperature *T*_*b*_ is isothermal.

**Fig. 2:**
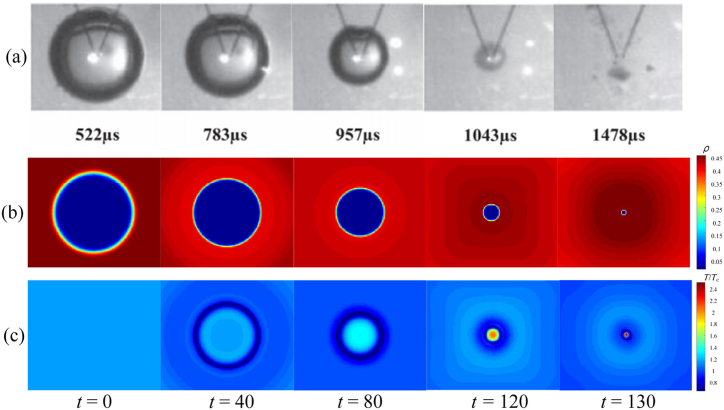
Comparison between experimental sequence images and LBM simulation results of single spherical cavitation bubble collapse. (a): Experimental results; (b): Density field evolution of LBM simulation; (c): Temperature field evolution of LBM simulation.

The comparison of experimental sequence image and LBM simulation results is illustrated in Fig. 2. The source of the experimental results is Ref. [55], the profile change of the single bubble collapse process was produced by high-voltage pulse discharge bubbles and captured high-speed cameras (Fig. 2(a)). Fig. 2(b) and (c) are the evolution process of density field and temperature field of multi-component cavitation bubbles simulated by LBM, respectively. The results show that the simulation results are in high agreement with the experimental results. In order to more accurately illustrate the feasibility of LBM simulation of cavitation bubble collapse, the following will be compared with the theoretical solution of cavitation bubble radius and temperature change.

### 3.2 Verification of Rayleigh-Plesset equation

The classic Rayleigh-Plesset (R–P) equation of cavitation bubble dynamic is

(23) $$\frac{p_{in}-p_{out}}{\rho_{l}}=R\ddot{R}+\frac{3}{2}\dot{R^{2}}+\frac{4\mu_{l}}{R}\dot{R}+\frac{2\sigma }{\rho_{l}R}$$

where $\dot{R}=dR/dt,\ddot{R}=d^{2}R/dt^{2}$, $\mu_{l}$ is the liquid dynamic viscosity which is related to the relaxation factor $\tau_{v}$, and σ refers to the surface tension. In this case, it is necessary to consider that the content of cavitation bubble is mixed gas. Assume that the gas characteristics in the bubble change exponentially

(24) $$p=p_{g}{\left(\frac{R_{0}}{R}\right)}^{3\gamma }$$

where *p*_*g*_ is the partial pressure of the impurity gas in the cavitation bubble, the gas adiabatic index *γ* is approximately constant. The R–P equation can be rewritten as Eq. (25) based on the above assumptions

(25) $$\frac{p_{v}-p_{out}\left(t\right)}{\rho_{l}}+\frac{p_{g}}{\rho_{l}}{\left(\frac{R_{0}}{R}\right)}^{3\gamma }=R\ddot{R}+\frac{3}{2}\dot{R^{2}}+\frac{4\mu_{l}R}{\dot{R}}+\frac{2\sigma }{\rho_{l}R}$$

**Fig. 3:**
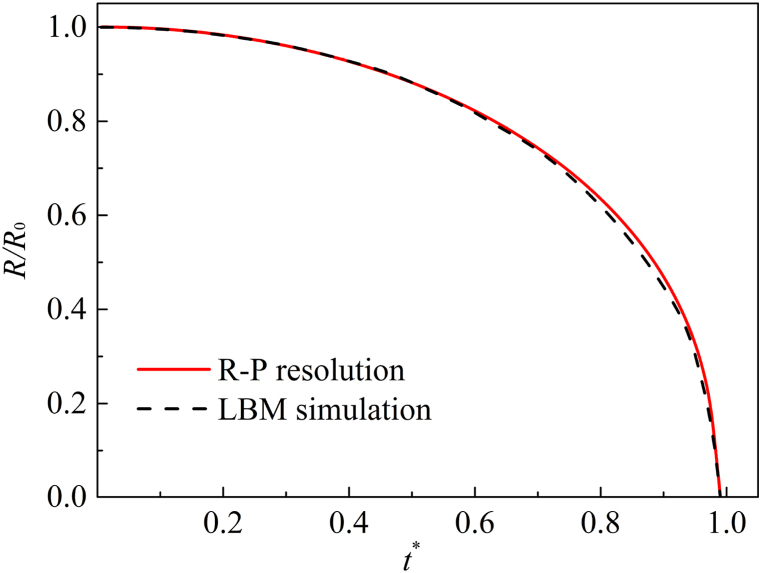
Comparison of bubble radius.

The initial outside pressure *p*_*out*_ = *p*_∞_ = 0.032768 is increased to ensure that the bubble collapses, and the pressure difference Δ*p* = *p*_∞_ *- p*_*b*_ = 0.000583. The parameters involved in LBM model, such as the pressure, density, radius, surface tension and viscosity, etc., are substituted into the R–P equation. The numerical calculation results are normalized, the LBM simulation is compared with the analytical resolution of R–P equation. Fig. 3 shows that the simulation results of LBM are consistent with those of R–P equation, which proved that the numerical model can successfully simulate MCMP flow.

### 3.3 Validation of adiabat law

The collapsing bubble produces extremely high temperature in the final stage. In this case, the collapsing process is considered to be adiabatic. According to adiabatic law, the equation for calculating instantaneous temperature of the bubble is [46].

(26) $$T=T_{\infty }{\left(\frac{R_{0}}{R}\right)}^{3\left(\gamma -1\right)}$$

where *T* is the instantaneous temperature of the bubble, and *T*_∞_ is the liquid temperature. *γ* represents the adiabatic index of gases. For an ideal gas adiabatic equation, the adiabatic index calculated by the following formula

(27) $$\gamma =\frac{c_{p}}{c_{v}}$$

As we know, the famous Meyer equation is written as *c*_*p*_ － *c*_*v*_ = *R*_*g*_. Thus, Eq. (27) can be converted as follows

(28) $$c_{v}=\frac{R_{g}}{\gamma -1}$$

**Fig. 4:**
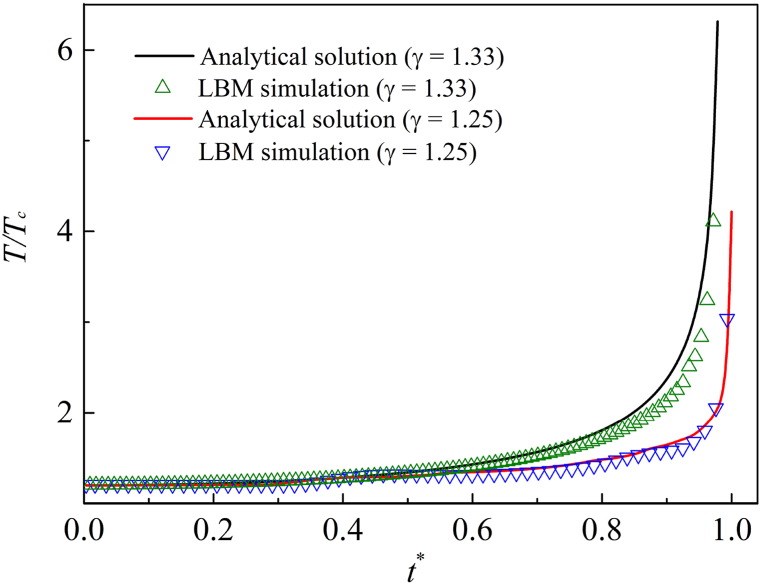
Verification of adiabat law.

In this section, the calculation model for temperature verification is still shown in Fig. 4. Two conditions, with adiabatic coefficients as 1.25 and 1.33 respectively, are simulated, and the initial liquid ambient temperature *T*_∞_ = 1.2*T*_*c*_. Other basic parameters are the same as section 3.2. The relationship between the bubble temperature and time steps can be further explored, and the timeline is normalized.

The LBM simulation results and the analytical solution are shown in Fig. 4. Under the same adiabatic index, the numerical simulation solution of LBM is in good agreement with the analytical solution. It demonstrates that the multicomponent thermal LBM is feasible to simulate MCMP flow.

## 4 Influence of gas adiabatic index on cavitation thermal effect

Studying the gas properties inside cavitation bubble is helpful to explore and discover the influence of the temperature dynamic change behavior and thermal effect during the bubble collapse. The adiabatic index depends on the type of gas, pressure and temperature. At a certain pressure and temperature, the adiabatic coefficient only related to the types of non-condensable gases inside bubble. In this work, the properties and types of gas is distinguished by changing the adiabatic index of the non-condensable gas.

### 4.1 Analysis of temperature distribution in flow field

**Fig. 5:**
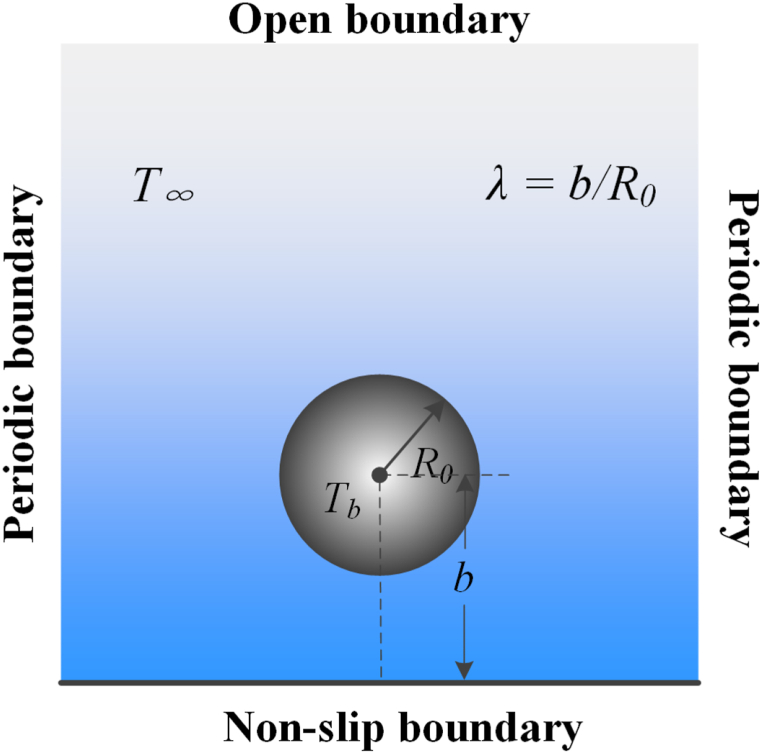
Computational domain.

In this simulation, a spherical bubble containing a two-gas mixture of non-condensable gas and water vapor is located in the central region of a gravity-free liquid. The domain size remains 301 × 301. The initial radius *R*_0_ = 60. Periodic boundary condition is imposed in the *x* and *y* directions. Top boundary is implemented as an open boundary, and the bottom wall is regarded as non-slip boundary condition. As shown in Fig. 5, the distance from the center to the wall is represented by the coefficient *b*. Position offset coefficient *λ* represents the ratio of *b* and *R*_0_. In the following simulation, the position offset coefficient *λ* = 1.3, and the initial ambient temperature *T*_∞_ = 1.2*T*_*c*_.

**Fig. 6:**
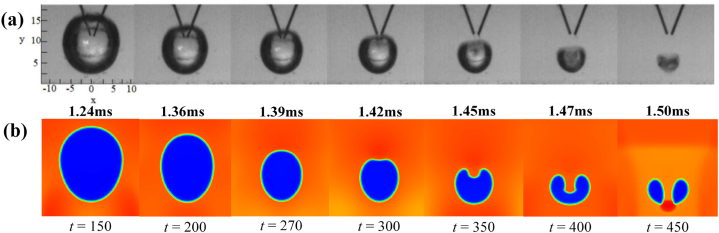
Comparison of bubble profile evolution between LBM and experimental results. (a): Experimental results of underwater pulsed discharge; (b) Density field of cavitation bubble.

Firstly, the bubble profile evolutions of cavitation bubble near a wall are compared with the experimental results of underwater pulsed discharge [57]. As shown in Fig. 6, the results are in good agreement. Furthermore, the temperature evolution process of cavitation bubble near the wall collapse under different *γ* are simulated.

**Fig. 7:**
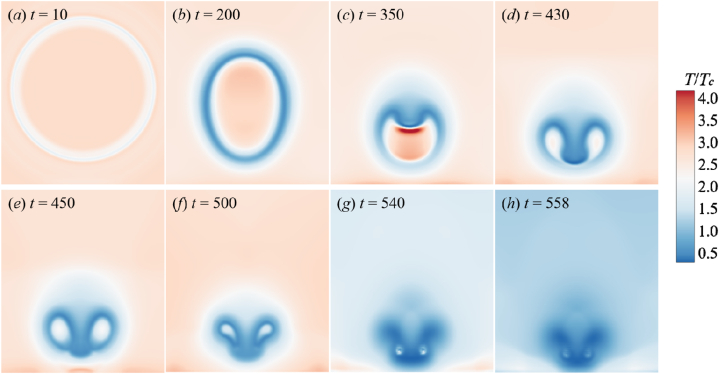
The temperature change process of the simulated flow field (*γ* = 1.33).

Fig. 7 shows the evolution of bubble temperature fields when the adiabatic coefficient of non-condensable gas is 1.33. From Fig. 7(a)–(c), it is obviously seen that the bubble temperature *T*_*b*_ increase as the bubble shrinks. A high-speed jet occurs above the bubble, causing the bubble to sag. As the degree of bubble depression increases, the bubble is about to penetrated by the jet. The bubble collapses for the first time in Fig. 7(d), which is called the first collapse. After the first collapse, the bubble is penetrated by the high-speed jet, which then crashing into the wall. Due to the pressure impact of jet, A high-temperature area, named “hot spot”, occurs on the wall Meantime, the movement of the surrounding fluid accelerates the contraction of the remaining annular bubble rapidly until it collapses completely, as shown from Fig. 7(e)–(g). In the final stage of collapse, as shown in Fig. 7(h). Also known as the bubble's second collapse, the temperature of the region near the collapse point will increase again and then gradually decrease.

The surrounding liquid temperature also changes during the collapse. The primary concern is the temperature variation in the liquid field around the bubble. In the initial stage, the surrounding liquid temperature exhibits a tendency opposite to the bubble temperature. The liquid temperature gradually decreases, especially at the place where the liquid flow velocity is larger (bubble depression), and the liquid temperature is relatively low. It is obvious that the liquid temperature is lower than the bubble temperature, which will affect the increase of the temperature in the bubble. Therefore, it makes the temperature in the post-collapse period hardly rise, and even has a downward trend.

**Fig. 8:**
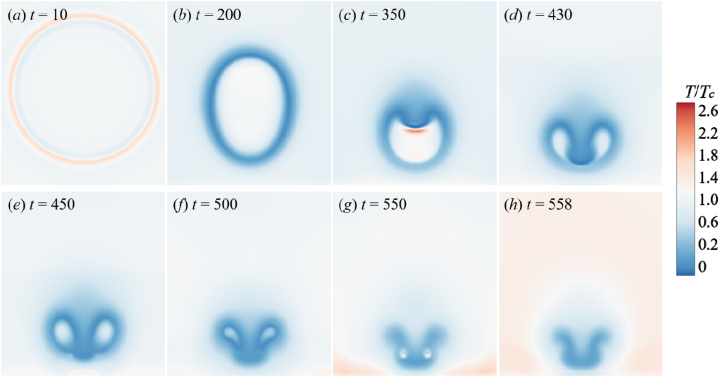
The temperature change process of the simulated flow field (*γ* = 1.25).

The temperature evolution is shown in Fig. 8 when the *γ* = 1.25. The bubble temperature increases as the bubble shrinks, but the temperature rise is not strong. Due to the accelerated condensation of vapor on the bubble surface, the temperature of the surrounding liquid region decreases (Fig. 8 (a) and (b)). Likewise, the high flow velocity at the top of the bubble creates a downward pressure that causes the bubble to depress. The temperature of the recessed portion has increased, as shown in Fig. 8(c). Fig. 8(d) is the bubble's first collapse. It can be seen from the legend that the bubble temperature is not much different from the external liquid temperature, the temperature inside bubble drops. As the first collapse progresses, the jet penetrates the bubble to form a downward shock wave. After the shock wave hit the wall, a region of high temperature is generated (Fig. 8(e)).

However, the temperature of this hot spot is significantly lower than the temperature of the hot spot formed in Fig. 7. During the second collapse (from Fig. 8(f)–(h)), due to the influence of the liquid flow velocity around the annular bubble, a high temperature area is formed in the collapse point, and the heats gradually transmit to the surrounding until dissipate.

**Fig. 9:**
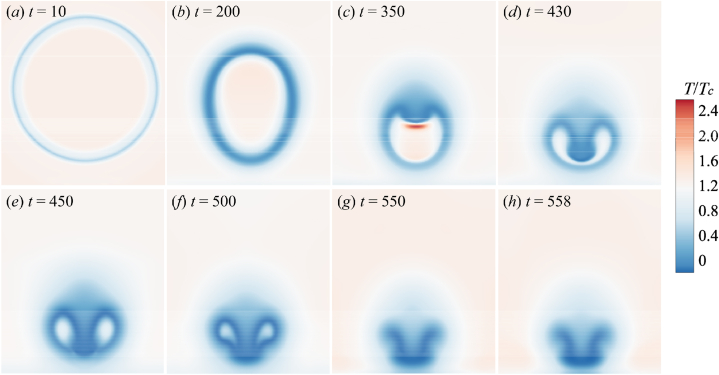
The temperature change process of the simulated flow field (*γ* = 1.16).

The temporal temperature distribution for *γ* = 1.16 is illustrated in Fig. 9. It can be observed that the evolution of the bubble's profile is consistent with Fig. 8, Fig. 9. This indicates that variations in the adiabatic index do not influence the pressure or velocity of the cavitation bubble itself and its surrounding fluid. Instead, these variations only alter the temperature distribution of the flow field. From Fig. 9(a)–(d), which correspond to the stage before the first collapse, the temperature inside the cavitation bubble gradually increases before decreasing. Fig. 9(e)–(h) depict the secondary collapse process of the bubble, revealing very low temperature inside the bubble and the surrounding liquid. Additionally, no prominent high-temperature hotspot forms on the solid bottom wall after the jet impacts the cavitation bubble. During this process, the wall temperature decreases instead (as indicated by the growing blue region).

From Fig. 7, Fig. 8, Fig. 9, the adiabatic index of the non-condensable gas will affect the temperature field evolution of the flow. For an actual non-condensable gas, this indicates that the *γ* has a relatively minor impact on the shape and dynamics of the bubble, primarily affecting the temperature distribution. Different adiabatic indices correspond to different types of gases, and when there are changes in gas type and properties, it will influence the temperature variations of the gas during the bubble collapse process, especially significantly affecting the bubble temperature and the temperature of the bottom wall. Therefore, the relationship between the *γ* and the internal bubble temperature as well as the wall temperature will be further analyzed in the following.

### 4.2 Cavitation bubble temperature

**Fig. 10:**
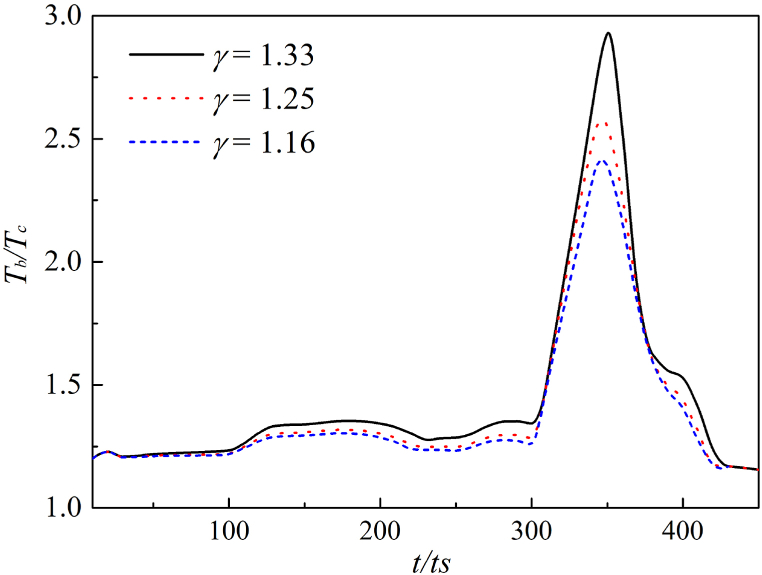
Maximum bubble temperature change in spherical bubble.

After the jet penetrates the cavitation bubble, the original spherical bubble becomes an annular. Therefore, we measured the inner temperature under two bubble forms. The results of the change of bubble temperature with time steps under different adiabatic coefficients *γ* are shown in Fig. 10, where *γ* is set as 1.33, 1.25 and 1.16 respectively.

Fig. 10 shows the temperature in the bubble initially increases slowly during the initial stage of collapse. After a jet is formed, *T*_*b*_ increases sharply. As the first collapse is completed, *T*_*b*_ gradually descends.

It can be found by comparing the temperature change curves for the three adiabatic coefficients. The curve corresponding to *γ* = 1.33 is the first to show an upward trend in temperature, and the magnitude of its temperature increase is the highest. As the value of the reduction *γ* decreases, the magnitude of the maximum temperature also decreases. It is clarified that the adiabatic index will affect the maximum temperature generated by the collapsing bubble. The higher the adiabatic index, the greater the degree of temperature increase.

**Fig. 11:**
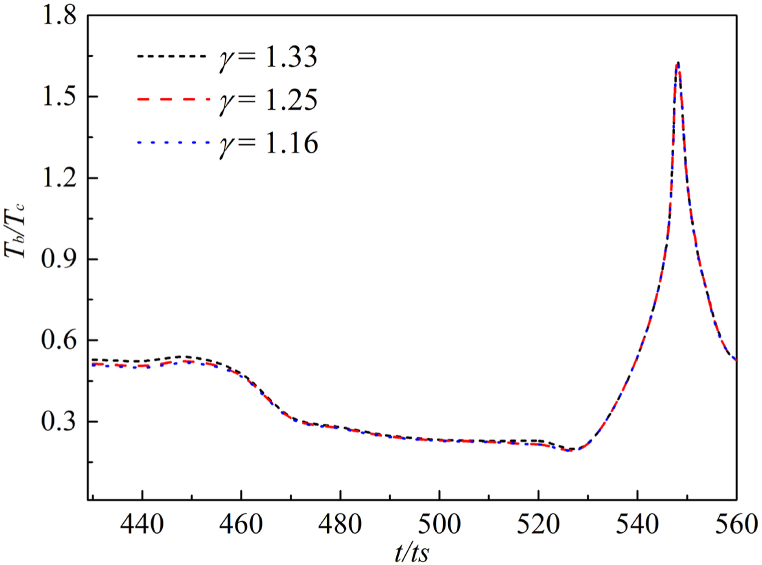
Maximum bubble temperature change in annular bubble.

In Fig. 11, the internal temperature changes of annular bubble after the second collapse under three adiabatic indexes. First, the temperature of the annular bubble undergoes a slow reduction process, then the effect of liquid rebound accelerates the flow velocity around the bubble, which accelerates the collapse of the bubble, resulting in high-temperature hot spot at the collapse point.

However, the consistence of three curves is shown in Fig. 11, indicating that the *γ* has little effect on the annular bubble collapse. The time if the second collapse is too short to make a difference, and the properties of non-condensable gas will not affect the final collapse temperature.

In a word, it is found that the difference of gas adiabatic index mainly affects the first stage of cavitation bubble collapse, i.e., the first collapse process, by LBM numerical simulation. While the *γ* has no significant effect on the second collapse.

### 4.3 Wall temperature and heat transfer

From the above analysis, it is comprehended that the gas adiabatic index affects the temperature of the flow field. Thus, it can be inferred that when gas properties various, the energy produced by the bubble collapse is different, thus the thermal effect on the solid surface will also be different. In order to verify this inference, the wall temperature is extracted and analyzed.

**Fig. 12:**
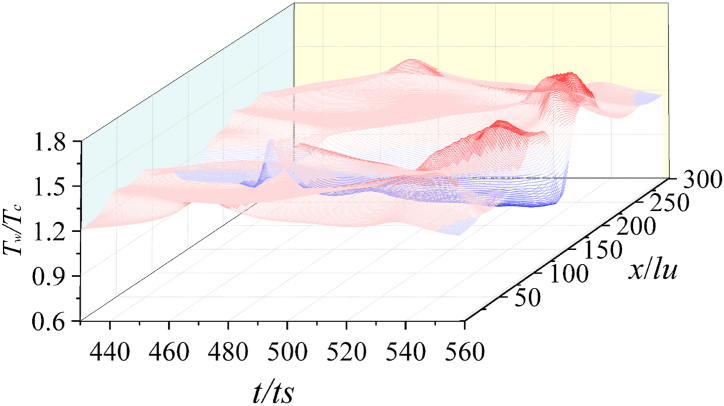
Time sequence of *T*_*w*_ (*γ* = 1.33).

Fig. 12 shows the time sequence of wall temperature *T*_*w*_ when *γ* = 1.33 and *λ* = 1.3. After the first collapse, the temperature of the center local area of the wall briefly increases, i.e., the hot spot area is formed. Then, the heat radiates and diffuses outwards. Initially, the temperature of the hot spot drops sharply, and the temperature of wall surface away from the central area gradually increases.

When the second collapse happens, the wall temperature has no obvious upward trend, indicating that the energy generated by the shock wave is weaker. The above simulation process shows that during the collapse stage, the temperature of the liquid area at the gas-liquid interface is lower, which reduces the temperature at the liquid-solid interface. The wall temperature of the central area decreases. On the contrary, the temperature in the peripheral area of the wall is higher due to the liquid outside the bubble is promoted by the secondary shock wave.

**Fig. 13:**
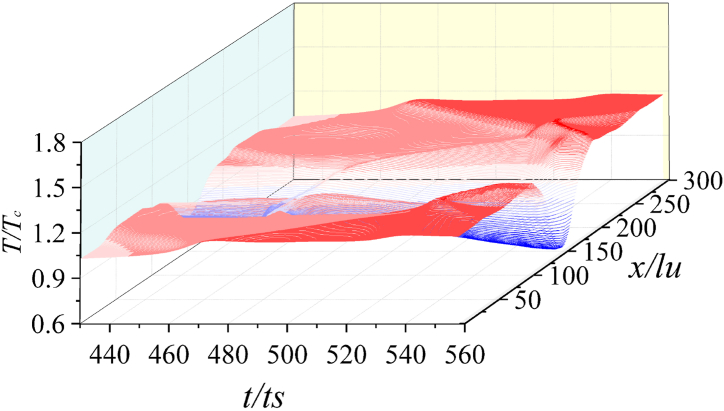
Time sequence of *T*_*w*_ (*γ* = 1.25).

Fig. 13 is the time sequence of *T*_*w*_ on the solid wall when *γ* = 1.25 and *λ* = 1.3. Significantly different from Fig. 12, the wall temperature does not increase obviously after collapse. Due to the decrease of the adiabatic index and the change of the gas properties, the energy produced by the first and second collapse is relatively low. In addition, the temperature of the liquid-solid interface directly under the bubble is lower than that of other regions of the solid wall. Owing to the acceleration of the peripheral liquid caused by the second collapse, the temperature of the solid wall region away from the central region is higher.

**Fig. 14:**
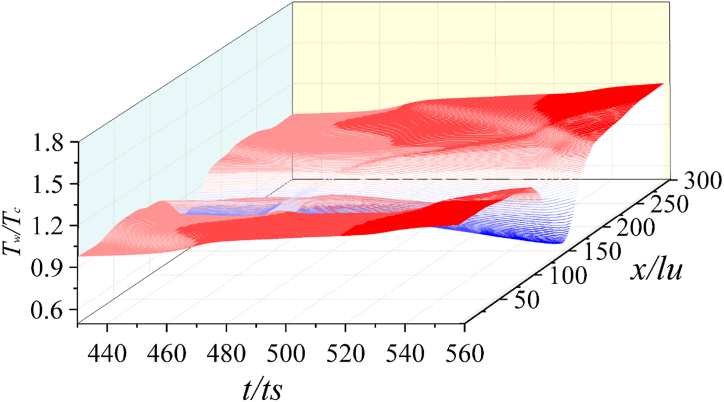
Time sequence of *T*_*w*_ (*γ* = 1.16).

Moreover, compared with Fig. 12, the temperature change amplitude in Fig. 13 is relatively stable. The curve trend in Fig. 14 is roughly the same as that in Fig. 13. The temperature of the central area of the wall is also lower than that of the surrounding wall.

**Fig. 15:**
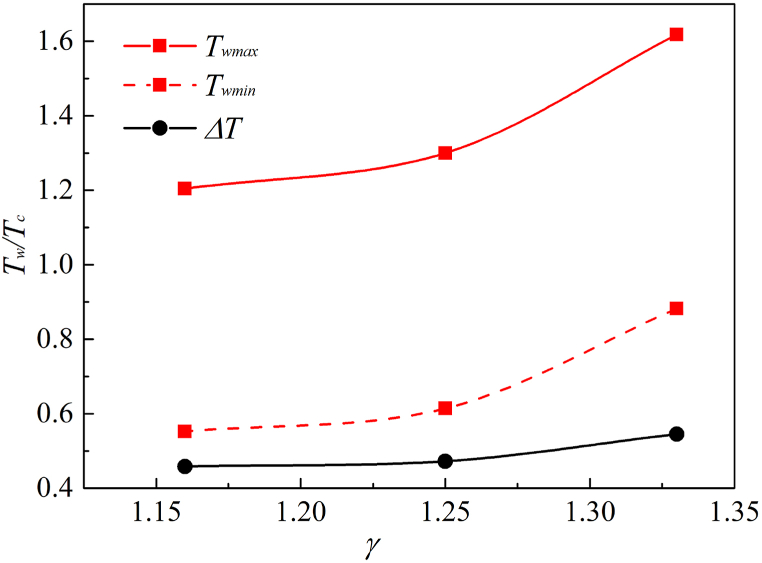
Change of *T*_*wmax*_, *T*_*wmin*_ and *ΔT* under different γ

In order to explain the relationship between wall temperature *T*_*w*_ and the adiabatic index *γ*, the maximum temperature *T*_*wmax*_ and the minimum temperature *T*_*wmin*_ on the wall are extracted quantitatively under different *γ*, and the relative temperature *ΔT= T*_*wmin*_/*T*_*wmax*_ is also analyzed, as shown in Fig. 15. It should be note that *ΔT* represents the relative change degree of the temperature in the high temperature area and low temperature of wall area. If *ΔT* is too large, it will cause a stronger effect between the thermal effect and the solid wall, and a greater temperature gradient between different areas of the solid surface.

From Fig. 15, the higher the *γ*, the higher the *T*_*wmax*_ (away from the center of the solid wall). The lower the *γ*, the lower the *T*_*wmin*_ (at the center of the solid wall). *ΔT* in the range *γ* = 1.25–1.15 is basically the same, indicating that if the coefficient is less than 1.25, the wall temperature changes more stably, and the *γ* is no longer the main factor affecting *T*_*w*_.

The above results show that due to the large thermal resistance of the bubble itself, the heat transfer in the wall area adjacent to the bubble surface becomes poor. While for the local area affected by the liquid jet, the heat transfer is enhanced. This phenomenon is mainly determined by the adiabatic index of the non-condensable gas. Theoretically, the adiabatic index of air is 1.40, and that of polyatomic gas is 1.29, or even less, such as 1.135 for dry saturated water vapor. Therefore, the increase of carbon dioxide (or water vapor) will reduce the adiabatic index of air.

In actual production and life, the non-condensable gas filled into cavitation bubble could be air, if we want to cool the surface of electronic components, which has a larger adiabatic coefficient. If we want the thermal effect of cavitation bubble collapse to be less effective, the carbon dioxide or water vapor gas can be increase to reduce the adiabatic index of air. In this way, the type of non-condensable gas can be selected according to different application sites, thus affecting the effect of cavitation thermal effect. Therefore, the cavitation technology can play a role in the field of heat transfer engineering.

## 5 Discussion and conclusion

In this paper, the influence of the gas adiabatic index on the thermodynamic process of cavitation bubble collapse is studied by an improved MCMP LB model. The temperature evolution of the flow field and the thermal effect on the wall are mainly investigated. Firstly, the developed DDF LBM model is verified by comparing the LBM numerical solution and the analytical solution obtained by solving R–P equation, which shows that LBM is correct for the cavitation case. Besides, the adiabatic law is verified. Under different adiabatic indexes, the internal temperature of the bubble simulated by LBM is compared with the theoretical solution, which further illustrates the effectiveness of the DDF LBM.

Then, we focus on the effect of gas properties on the temperature of the cavitation flow field. The evolution of temperature fields is simulated by adjusting the adiabatic index *γ*. Several interesting phenomena are found: (1) the internal temperature of the bubble will undergo a process of increasing and decreasing, and the temperature will increase during the two collapse processes of the bubble. (2) the value of the gas adiabatic index *γ* will affect the first collapse. The larger the *γ*, the higher the maximum bubble temperature. however, the *γ* will not have a special impact on the process of the second collapse. (3) the temperature of the liquid near the bubble surface will decrease as the collapse processes, especially the liquid temperature above the depression area of the bubble. The formation of low temperature regions in turn affects the bubble temperature. (4) the temperature resistance of the bubble itself is relatively large, thus the temperature of the liquid-wall area under the bubble is lower, while the temperature in other areas affected by the liquid jet is relatively high. (5) the gas adiabatic index *γ* is able to affect the maximum temperature *T*_*wmax*_, the minimum temperature *T*_*wmin*_ and the relative temperature *ΔT*.

The present results indicate that the variation among the *γ*, the bubble temperature *T*_*b*_ and the wall temperature *T*_*w*_. The analysis reveals how the *γ* influences the temperature distribution and thermal effects. By manipulating the type and properties of non-condensable gases inside cavitation bubbles, it's possible to control and optimize the thermal effects of bubble collapse. This insight has implications for cooling or heating surfaces in various engineering applications, such as electronic component cooling and environmental protection refrigeration. Future work will focus on the development of 3D models and the research on LBM algorithm acceleration.

## Data availability statement

Data will be made available on request.

## CRediT authorship contribution statement

**Yu Yang:** Conceptualization, Data curation, Formal analysis, Investigation, Methodology, Project administration, Resources, Software, Writing – original draft. **Minglei Shan:** Funding acquisition, Methodology, Supervision, Writing – review & editing. **Xuefen Kan:** Funding acquisition, Validation, Visualization. **Kangjun Duan:** Methodology, Software, Writing – review & editing. **Qingbang Han:** Supervision, Writing – review & editing, Funding acquisition. **Yue Juan:** Funding acquisition, Writing – review & editing.

## Declaration of competing interest

The authors declare that they have no known competing financial interests or personal relationships that could have appeared to influence the work reported in this paper.
